# Expression dynamics of WOX homeodomain transcription factors during somatic embryogenesis in *Liriodendron* hybrids

**DOI:** 10.48130/FR-2023-0015

**Published:** 2023-06-13

**Authors:** Xiaofei Long, Jiaji Zhang, Dandan Wang, Yuhao Weng, Siqin Liu, Meiping Li, Zhaodong Hao, Tielong Cheng, Jisen Shi, Jinhui Chen

**Affiliations:** 1 State Key Laboratory of Tree Genetics and Breeding, Co-Innovation Center for Sustainable Forestry in Southern China, Nanjing Forestry University, Nanjing 210037, China; 2 Key Laboratory of Forest Genetics & Biotechnology of Ministry of Education, Co-Innovation Center for Sustainable Forestry in Southern China, Nanjing Forestry University, Nanjing 210037, Jiangsu, China

**Keywords:** WOX family, *Liriodendron*, Somatic embryogenesis

## Abstract

The relict woody plant genus *Liriodendron* contains two endangered species, *Liriodendron chinense* and *Liriodendron tulipifera*. Understanding the molecular mechanisms involved in early embryo development is important for horticultural and ecological research, particularly for the development of improved somatic embryogenesis systems. However, the specific molecular processes underlying embryogenesis in these species remain largely unexplored. To address this, we investigated expression of the *WOX* ( *WUSCHEL-related homeobox*) gene family of transcription factors throughout somatic embryogenesis. We confirmed expression of eight out of 11 novel candidate *LcWOX* genes in *L.chinense* using qRT-PCR and examined spatiotemporal expression patterns of the expressed genes using stable reporter lines that had been transformed with different *LcWOX* promoters driving GUS expression. We observed embryo developmental stages and expression patterns that broadly correlated with those reported for *Arabidopsis* somatic embryogenesis. *LcWUS* was weakly expressed during the transition stage and was predominantly restricted to the apical meristem. *LcWOX5* was specifically expressed in the root meristem and restricted to the cotyledons thereafter, and *LcWOX4* expression was restricted to the vascular tissue of cotyledonary embryos. In contrast, *LcWOX9* was expressed in the embryonic callus and the entire embryonic cell mass, then became restricted to the basal cells, indicating a potential role in regulating embryonic maintenance. Our findings provide insights into spatiotemporally specific *WOX* transcription and shed new light on potential functions of *WOX* genes during *Liriodendron* somatic embryogenesis.

## Introduction

*Liriodendron*, a genus of the Magnoliaceae family, contains just two sister species: *Liriodendron chinense* (Hemsl.) Sarg. and *Liriodendron tulipifera* L. *Liriodendron* is an excellent ornamental tree for landscaping, because of its straight trunk, conical crown, distinctive leaf shape, and tulip-shaped flowers ^[ 1] ^. *Liriodendron chinense* is found sporadically from central-western and southern China to northern Vietnam, whereas *Liriodendron tulipifera* is distributed extensively across eastern North America ^[ 2] ^. The sister species possess characteristics typical of both monocots and eudicots. Their reproductive organs have monocot features such as flower parts in multiples of three and monocolpate pollen grains, whereas their vegetative organs have eudicot features like netted venation and paired cotyledons. Thus, the evolutionary position of the Magnoliaceae family, represented by *Liriodendron*, is highly controversial. As in other hardwood species, the study of embryogenesis in *Liriodendron* has always been challenged by the difficulty of obtaining materials from nature, the extended juvenile period, and the constraints of the developmental cycle ^[ 3] ^.

Recent research has revealed intriguing similarities between the embryogenesis process of *Liriodendron* and that of the model dicot *Arabidopsis thaliana* ^[ 4] ^. In *Arabidopsis*, the zygote undergoes several rounds of asymmetric division, leading to a series of well-defined morphological stages including the pre-embryo, globular, heart-shaped, transition, torpedo, and cotyledon embryo stages. In contrast to *Arabidopsis,* the further cell division patterns of the apical and basal cell lineages appear random and are less stereotypic in grasses ^[ 5] ^, which lack a morphologically typical globular embryo and heart-shaped embryo stage.

Little is currently known about the molecular mechanisms of embryogenesis in *Liriodendron*. In comparison to animal cells, plant cells show considerable plasticity, and the totipotency and pluripotency of plant cells facilitate their regeneration ^[ 6] ^. During *in vitro* culture, hormones trigger plant tissues and cells to regenerate into new organs or entire plants. In general, plant regeneration can be categorized into three types: tissue reparation, *de novo* organogenesis—including *de novo* root regeneration and shoot regeneration—and somatic embryogenesis ^[ 7] ^.

Somatic embryogenesis (SE) is an *in vitro* process in which plant somatic cells are induced to form embryos without meiotic division and double fertilization ^[ 8] ^. There are two types of SE: direct and indirect. Direct SE is a process in which somatic cells are stimulated to form embryos directly without the need for an intervening callus stage. On the other hand, indirect SE involves the formation of a callus structure from somatic cells, followed by the formation of embryogenic cells from the callus. Indirect SE is a slower process than direct SE, but it can be used for plants that are difficult to regenerate directly from somatic cells. Both direct and indirect somatic embryogenesis are effective ways to propagate woody plants *in vitro* ^[ 9] ^. Compared with traditional breeding methods like seeding, grafting, and layering, somatic embryogenesis offers advantages such as short incubation periods, higher reproduction efficiency, and reduced seasonal influence ^[ 10] ^.

Within the plant kingdom, somatic embryogenesis in a number of species appears to involve morphological stages identical to those of zygotic embryogenesis (i.e., globular, transition-stage, heart-shaped, and cotyledon embryo stages). It is likely that the molecular mechanisms regulating the transitions between these developmental stages, such as activation of specific genes and genetic pathways that regulate cell fate and pattern formation, are similar ^[ 11] ^.

Because of its single-cell origin and high reproduction coefficient, the somatic embryogenesis system in *Liriodendron* enables rapid and effective propagation and is critical for genetic transformation and genome editing ^[ 12] ^. In *Liriodendron*, embryogenic callus is induced from immature zygotic embryos by addition of exogenous auxin (2,4-D) to 1/2 Murashige and Skoog (MS) medium. Somatic embryos can then be produced from the embryogenic callus by addition of sucrose to raise the osmotic pressure and removal of exogenous auxin. Abscisic acid is used to prevent the formation of abnormal embryos ^[ 13] ^.

The *WUSCHEL* ( *WUS*) *-related homeobox* ( *WOX*) gene family is a class of homeodomain (HD) transcription factors involved in embryogenesis and lateral organ development in plants ^[ 14] ^. The original family member, *WUS*, is essential for stem cell maintenance in the shoot apical meristem ^[ 15] ^, and auxin-induced *WUS* expression is necessary for embryonic stem cell renewal during somatic embryogenesis in *Arabidopsis* ^[ 16] ^. Upon cellular reprogramming, direct activation of the early embryonic patterning genes *WOX2* and *WOX3* either increases the rate of somatic embryogenesis or improves somatic embryo development ^[ 17] ^.

We set out to understand the potential functions of *WOX* genes during somatic embryogenesis of *Liriodendron* through quantitative expression and phylogenetic analyses followed by assessment of transcriptional reporter lines during successive developmental stages. The results provide foundational information that is crucial for understanding the function of *WOX* genes during *Liriodendron* somatic embryogenesis.

## Results

### Somatic embryogenesis in the *Liriodendron* hybrids

**Figure 1:**
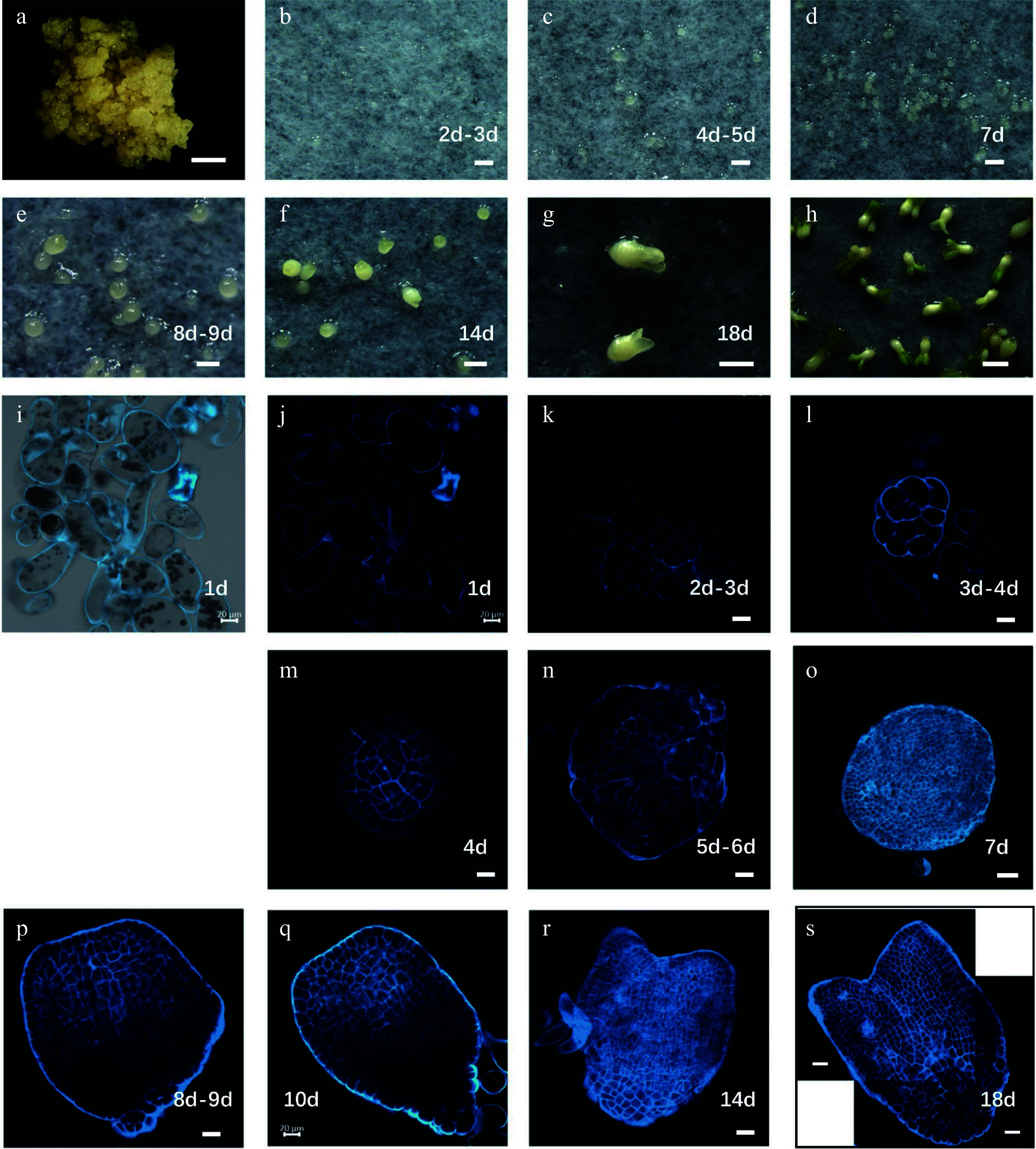
Developmental stages of *Liriodendron* hybrids somatic embryos. (a) Embryonic callus; (b) embryos after 2–3 d on embryo induction medium (EIM); (c) pre-globular embryo after 4–5 d on EIM; (d) globular embryo after 7 d on EIM; (e) transition stage embryo after 8–9 d on EIM; (f) heart-shaped embryo after 14 d on EIM; (g) cotyledon embryos after 18 d on EIM; (h) plantlets after cultivation in the light; (i–s) different stages of somatic embryos observed by confocal microscopy with SCRI 2200 staining. Scale bars: (a)–(d) 200 μm; (e)–(h) 100 μm; (i)–(n) 20 μm; (o) 50 μm; (p)–(s) 20 μm.

To induce somatic embryos within the *Liriodendron* hybrids that were produced firstly by Professor Peizhong Ye who successfully implemented hybrid breeding in *Liriodendron* ^[ 18] ^, immature zygotic embryos containing endosperm were collected and incubated on callus induction medium (CIM). Embryogenic callus was then initiated from immature embryos after at least two rounds of subculturing, which lasted about 2 months ( Fig. 1a). Embryogenic callus is beige in color and dense in texture, whereas non-embryonic callus is white ^[ 19] ^. After 30 min of suspension culture in liquid CIM, the cultures were passed through successive stainless-steel sieves, and the suspended cells were spread on sterile filter paper and placed on embryo induction medium (EIM). Tiny cell groups could be observed on the EIM after 2–3 d of incubation ( Fig. 1b). After 5 d, typical globular embryos were observed ( Fig. 1c), and after 7 d, globular embryos with a diameter of approximately 80–150 μm had developed ( Fig. 1d). Heart-shaped and cotyledon embryos were also observed on subsequent days ( Fig. 1e– h).

To study somatic embryo morphology at the cellular level, we stained embryos of different stages with SCRI Renaissance 2200 (SR2200) ^[ 20]^ and examined them using a confocal microscope. We observed single cells 1 d after induction, demonstrating that the *Liriodendron* somatic embryos were of single-cell origin ( Fig. 1i– j). The cells divided rapidly and progressively transformed into pre-globular embryos ( Fig. 1k– m). Typical globular embryos were observed after 6–7 d of induction, although their shoot and root poles were difficult to distinguish morphologically ( Fig. 1n– o). After 10 d of induction, the embryos entered the transition stage when shoot and root poles were clearly distinguishable ( Fig. 1p– q). After 14 d of induction, heart-stage embryos with clear bilateral symmetry had formed ( Fig. 1r). The fundamental embryonic features of the plant body were established by the torpedo stage (after 18 d of induction) ( Fig. 1s). Although the somatic embryos did not exhibit invariant patterns of cell division, they followed the same morphological developmental route as *Arabidopsis* zygotic embryos, indicating that *Liriodendron* somatic embryogenesis is very similar to the zygotic embryogenesis of dicot plants ^[ 21] ^.

### Identification of *LcWOX* gene family members

**Figure 2:**
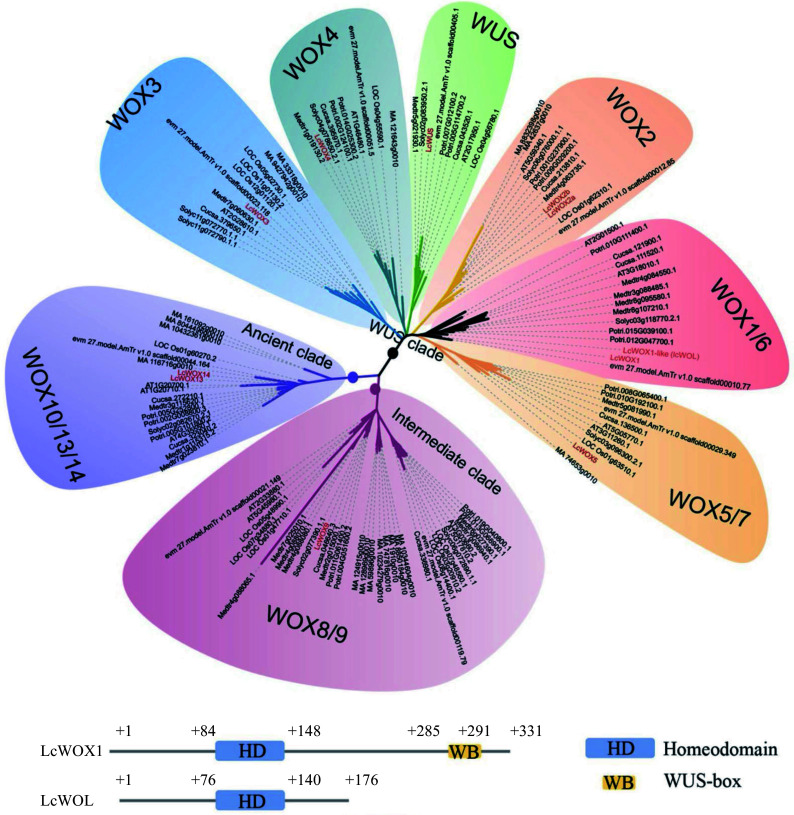
*LcWOX* genes were grouped into three distinct, well-supported clades. WOX orthologs come from fully sequenced genomes of five eudicot species, *Arabidopsis thaliana(AT), Medicago truncatula (Medtr),* *Cucumis sativus (Cucsa), Solanum lycopersicum(Solyc)* and *Populus trichocarpa (Potri)*, as well as a monocot species: *Oryza sativa(LOC Os). WOX* gene sequences from the gymnosperm *Picea abies(MA)* and basal Magnoliophyte, *Amborella trichopoda(evm27.model.AmTr.V1.0)* also have been included.

The WOX family plays an important role in early phases of embryogenesis. Differential expression of *WOX* genes affects the cell fate of early embryonic cells, including the maintenance of cell division and differentiation, and then promotes organogenesis ^[ 22] ^. Protein sequences encoded by the 15 *Arabidopsis* *WOX* genes were used as queries to search for candidate *LcWOX* genes in the *L. chinense* genome. We identified 11 candidate *LcWOX* genes with conserved homeodomains and classified them into three well-supported clades by phylogenetic analysis ^[ 23] ^. To enhance the resolution of our phylogenetic tree, we included *WOX* genes from additional species: one monocot ( *Oryza sativa* L.), three dicots ( *Solanum lycopersicum*, *Medicago truncatula*, *Populus trichocarpa*), and representatives of basal angiosperms and gymnosperms ( *Amborella trichopoda* and *Picea abies*) ( Fig. 2). The *WUS* clade contained the most members (eight out of 11 *LcWOX* genes), whereas the intermediate and ancient clades contained only one and two members, respectively ( Fig. 2, highlighted in red).

In *Arabidopsis*, *WOX7* from the WUS clade and *WOX13* and *WOX14* from the ancient clade lack the WUS-box structure, whereas all other *WOX* genes contain this motif ^[ 22] ^. Here, we identified one *LcWOX* gene that lacks the WUS-box but whose homeodomain amino acid sequence bears a striking resemblance to that of LcWOX1. We therefore designated this gene *LcWOX1-LIKE* ( *LcWOL*) ( Fig. 2).

### Expression analysis of the *LcWOX* genes in *Liriodendron* by qRT-PCR

We next analyzed the expression of 11 *LcWOX* genes in multiple *Liriodendron* tissues by qRT-PCR. *LcWUS* transcript levels were low in mature tissues such as the root and leaf but high in immature floral organs (Supplemental Fig. S1a). *LcWOX1* was strongly expressed in the leaf, shoot, and floral organs of *L. chinense* but was not detected in the roots (Supplemental Fig. S1b). There are two copies of *LcWOX2*, *LcWOX2A* and *LcWOX2B.* *LcWOX2A* showed extremely high expression in roots and modest expression in all other tissues (Supplemental Fig. S1c). This expression pattern was entirely different from that of *AtWOX2,* which is expressed in the zygote and embryo proper during embryogenesis ^[ 24] ^. *LcWOX2B* differed from *LcWOX2A* in loss of one amino acid from the middle of the protein sequence. It was therefore difficult to design suitable primers that could discriminate between these two genes, and we did not further investigate *LcWOX2B* expression in individual tissues.

*LcWOX3* expression was detected in the shoot and floral organs but not in the roots (Supplemental Fig. S1d) *. LcWOX4* was highly expressed in roots and stems but showed lower expression in other tissues, such as leaves and floral organs (Supplemental Fig. S1e). This expression profile was similar to that reported for *WOX4* in *Arabidopsis* ^[ 25] ^, in which the intercellular TDIF-TDR-WOX4 signaling pathway regulates cambium cell division, particularly in the root and stem ^[ 26] ^. Although *LcWOX5* expression was detected in the roots, it was higher in the floral organs (Supplemental Fig. S1f). Judging from this expression pattern, it is possible that *LcWOX5* functions in the quiescent center as an 'organizing center' that imparts a stem cell state to neighboring cells and also mediates the development of other organs in *Liriodendron*. *LcWOX9* was expressed in all examined tissues except for leaves. It was highly expressed in the bud, gynoecium, and stem but was less abundant in roots, stamens, and petals (Supplemental Fig. S1g). *LcWOX13* was barely detectable in any tissue, suggesting that it may be a pseudogene like *AtWOX10* ^[ 27]^ *. LcWOX14* was highly expressed in reproductive organs such as the gynoecium but showed lower expression in vegetative organs (Supplemental Fig. S1h).

### Expression of *LcWOX* genes during somatic embryogenesis in *Liriodendron*

To investigate expression patterns of the *LcWOX* genes during somatic embryogenesis (SE) in the *Liriodendron* hybrids, we first performed qRT-PCR analysis (Supplemental Fig. S2) and then generated 10 stable transcriptional reporter lines in which GUS expression was driven by different *LcWOX* promoter fragments, each > 3 kb in length. These reporter lines were used to reveal the expression patterns of 10 *LcWOX* genes (excluding *LcWOL*) at different stages of SE.

### A. Expression patterns of *LcWUS*-clade genes during somatic embryogenesis

**Figure 3:**
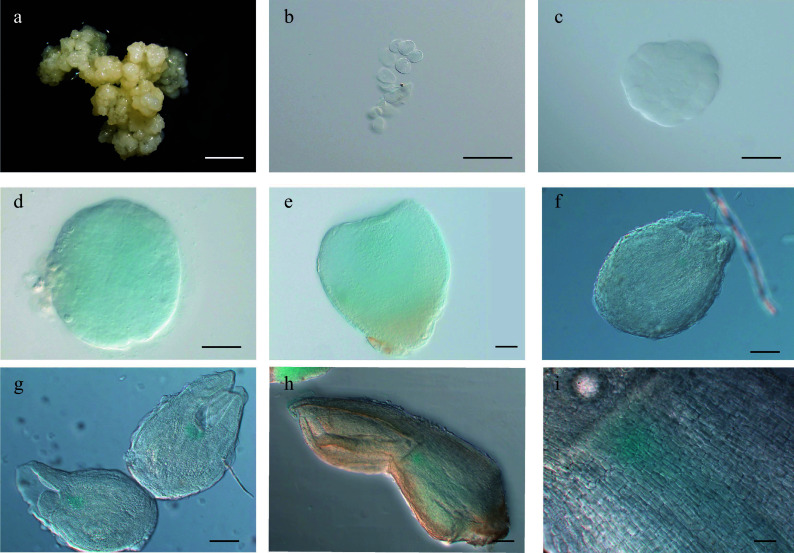
Expression pattern of *LcWUS*_*pro*_: *GUS* during somatic embryogenesis in *Lirodendron*. (a) – (c) *LcWUS*_*pro*_: *GUS* was not expressed in (a) embryonic callus, (b) single cells after 1 d on induction medium (IM), or (c) the pre-globular embryo after 4 ds on IM. (d), (e) *LcWUS*_*pro*_: *GUS* was weakly expressed in the (d) globular embryo and (e) heart-shaped embryo. (f) – (i) *LcWUS*_*pro*_: *GUS* was expressed in a tissue-specific manner in the (f) late heart-shaped embryo, (g) torpedo embryo, and (h) mature cotyledon embryo. (i) Magnification of the OC area in (h). Scale bars: (a) 2000 μm; (b) 100 μm; (c) – (e) 50 μm; (f), (g) 100 μm; (h) 200 μm; (i) 500 μm.

In *Arabidopsis, WUS* functions to maintain the identity of stem cells and is expressed in the inner apical cells of the 16-cell-stage embryo. Its expression in the shoot apical meristem is known to be confined to the organizing center (OC) ^[ 28]^ post-embryonically. *AtWUS* is induced in embryonic calli (EC) before SE can be recognized morphologically ^[ 16] ^. Here, *LcWUS* was highly expressed in the proembryo, but its expression declined during subsequent embryonic stages (Supplemental Fig. S2a). We did not detect clear GUS staining of EC in the *LcWUS*_*pro*_: *GUS* reporter line ( Fig. 3a), but the *LcWUS*_*pro*_: *GUS* signal was clearly visible in globular, heart-shaped, and cotyledon embryos ( Fig. 3d– i). From the late heart stage onwards, the *LcWUS*_*pro*_: *GUS* signal was detected specifically in the OC of the shoot apical meristem ( Fig. 3f– i). By contrast, *35S*_*pro*_: *GUS* showed broad, non-tissue-specific expression during SE (Supplemental Fig. S3).

**Figure 4:**
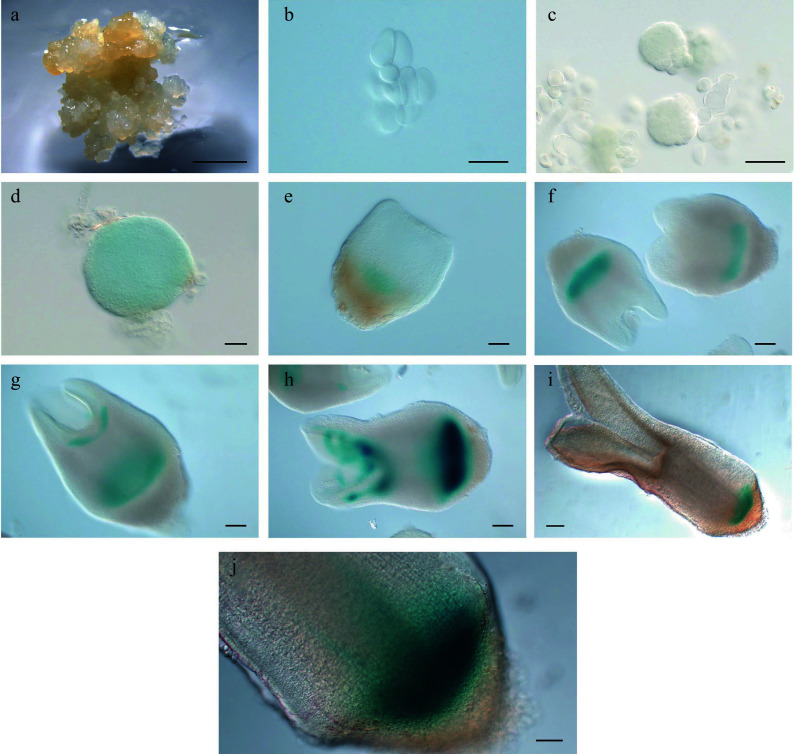
Expression pattern of *LcWOX5*_*pro*_: *GUS* during somatic embryogenesis in *Liriodendron*. (a)−(c) *LcWOX5*_*pro*_: *GUS* was not expressed in (a) embryonic callus, single cells after one day on (b) induction medium (IM), and (c) pre-globular embryos after four days on IM. (d) *LcWOX5*_*pro*_: *GUS* was expressed in the globular embryo. (e)−(j) *LcWOX5*_*pro*_: *GUS* was expressed in a tissue-specific manner in the (e) transition-stage embryo, (f), (g) late heart-shaped embryo, (h) torpedo embryo, (i) mature cotyledon embryo, and (j) plantlet root apical meristem of. Scale bar: (a) 2,000 μm; (b)−(e) 50 μm; (f)−(h) 100 μm; (i) 200 μm; (j) 100 μm.

*AtWOX5* is important for the maintenance of *Arabidopsis* root apical meristem ^[ 29] ^. After apical-basal polarity is established, the SE body plan is further refined by the initiation of the shoot and root apical meristems and cotyledons ( Fig. 1f). We observed that the expression of *LcWOX5* increased dramatically in heart-shaped embryos and peaks at the torpedo stage (Supplemental Fig. S2f). *LcWOX5*_*pro*_: *GUS* was actively expressed from globular embryos onwards ( Fig. 4d− j). At the early heart-shaped embryo stage, the *LcWOX5*_*pro*_: *GUS* signal could be detected in a small group of cells in the root ( Fig. 4e). Notably, at this stage, the expression of *LcWUS*_*pro*_: *GUS* was not QC specific ( Fig. 3e). In addition to expression in the root tip, we observed a GUS signal in the cotyledons ( Fig. 4g, h). The cotyledon expression of *LcWOX5*_*pro*_: *GUS* appeared to be the strongest during the late torpedo stage of SE, and then gradually decreased or disappeared in the plantlet ( Fig. 4i).

**Figure 5:**
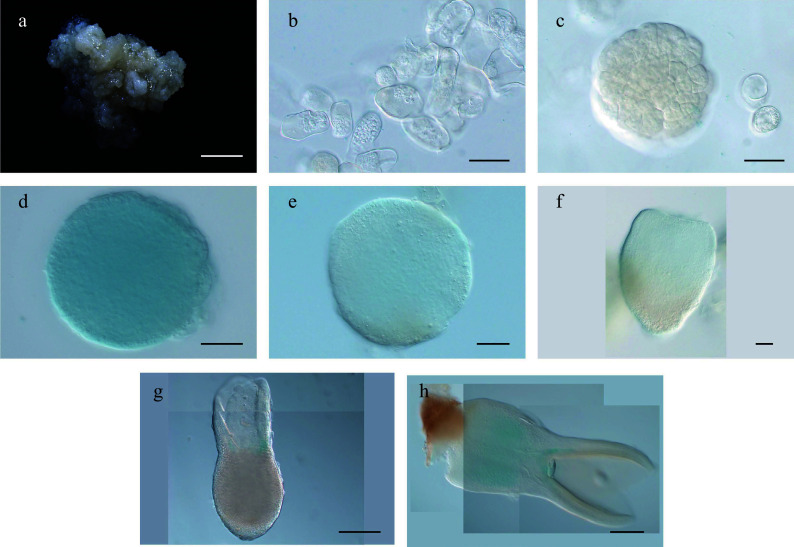
Expression pattern of *LcWOX1*_*pro*_: *GUS* during somatic embryogenesis in *Liriodendron*. (a)–(c) *LcWOX1*_*pro*_: *GUS* was not expressed in (a) embryonic callus, (b) single cells after 1 d on induction medium (IM), or the (c) pre-globular embryo. (d)–(f) *LcWOX1*_*pro*_: *GUS* was weakly expressed in the (d), (e) globular embryo and (f) transition-stage embryo. (g) *LcWOX1*_*pro*_: *GUS* was expressed at the base of the cotyledon in the torpedo embryo. (h) *LcWOX1*_*pro*_: *GUS* was expressed in the cotyledon and hypocotyl of the mature cotyledon embryo. Scale bars: (a) 2,000 μm; (b)–(e) 50 μm; (f)–(h) 200 μm.

In *Arabidopsis*, *WOX1* expression is confined to the initiating vascular primordia of cotyledons after the heart stage ^[ 30] ^. Our qRT-PCR results revealed that *LcWOX1* was not expressed during the early phase of SE but was expressed from the heart-shaped embryo stage until the cotyledon embryo stage and at an even higher level in the regenerated plantlet (Supplemental Fig. S2b). *LcWOX1*_*pro*_: *GUS* was inactive during the early stages of SE ( Fig. 5a– c), but its expression was observed from the globular to cotyledon stages ( Fig. 5d– h), particularly at the base of cotyledons and in the hypocotyl ( Fig. 5g, h).

**Figure 6:**
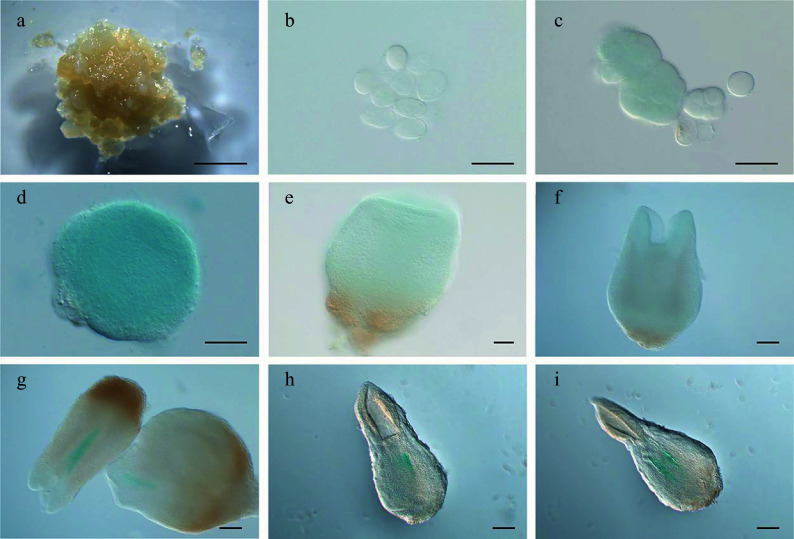
Expression pattern of *LcWOX4*_*pro*_: *GUS* during somatic embryogenesis in *Liriodendron*. (a), (b) *LcWOX4*_*pro*_: *GUS* was not expressed in (a) embryonic callus or (b) single cells after 1 d on induction medium (IM). (c) *LcWOX4*_*pro*_: *GUS* was weakly expressed in the (d) pre-globular embryo but highly expressed in the globular embryo. (e), (f) *LcWOX4*_*pro*_: *GUS* was weakly expressed in the (e) transition-stage embryo and(f) late heart-shaped embryo. (g)–(i) *LcWOX4*_*pro*_: *GUS* was expressed in a tissue-specific manner in the (g) torpedo embryo and (h), (i) cotyledon embryo. Scale bars: (a) 2,000 μm; (b)–(e) 50 μm; (f)–(h) 200 μm.

Like *LcWOX1*, *LcWOX4* was expressed at low levels in embryogenic callus and early stages of SE, but its expression increased throughout SE (Supplemental Fig. S2e). Compared with *LcWOX1*, *LcWOX4* showed a more pronounced decrease in expression in regenerated plantlets. *WOX4* transcription has previously been detected in the vasculature and during lateral organogenesis of *Arabidopsis*, *Populus*, and tomato ^[ 31] ^. Here, *LcWOX4*_*pro*_: *GUS* was not expressed or showed very weak and diffuse expression in the embryo before the early torpedo stage ( Fig. 6a– f); it was expressed specifically in the vascular cells at the later torpedo stage ( Fig. 6g– i).

Expression patterns inferred from the reporter lines differed from those measured by qRT-PCR for some *LcWOX* genes from the WUS clade (Supplemental Fig. S2). For example, we detected high levels of *LcWOX2A* transcripts in EC by qRT-PCR (Supplemental Fig. S2c). *LcWOX2A* expression was 0.5-fold lower in proembryos than in the EC, increased in globular and heart-shaped embryos, and declined again at later stages. However, the GUS signals of *LcWOX2B*_*pro*_: *GUS* lines showed no spatiotemporal specificity or changes during SE (data not shown). Similarly, qRT-PCR results indicated that *LcWOX3* expression was higher at later stages than early stages of SE (Supplemental Fig. S2d), but the *LcWOX3*_*pro*_: *GUS* reporter lines showed a different expression pattern, with a weak and diffuse GUS signal detected only at later stages (data not shown). This discrepancy may have been caused by the use of 3,470-bp and 3,479-bp promoter fragments respectively that lacked some essential or long-range elements, as we could not use a very long promoter. Further study is needed to determine the appropriate promoter length and/or to locate additional fragments in the genome that regulate expression.

### B. Expression patterns of the intermediate clade genes *WOX8/9* during somatic embryogenesis

**Figure 7:**
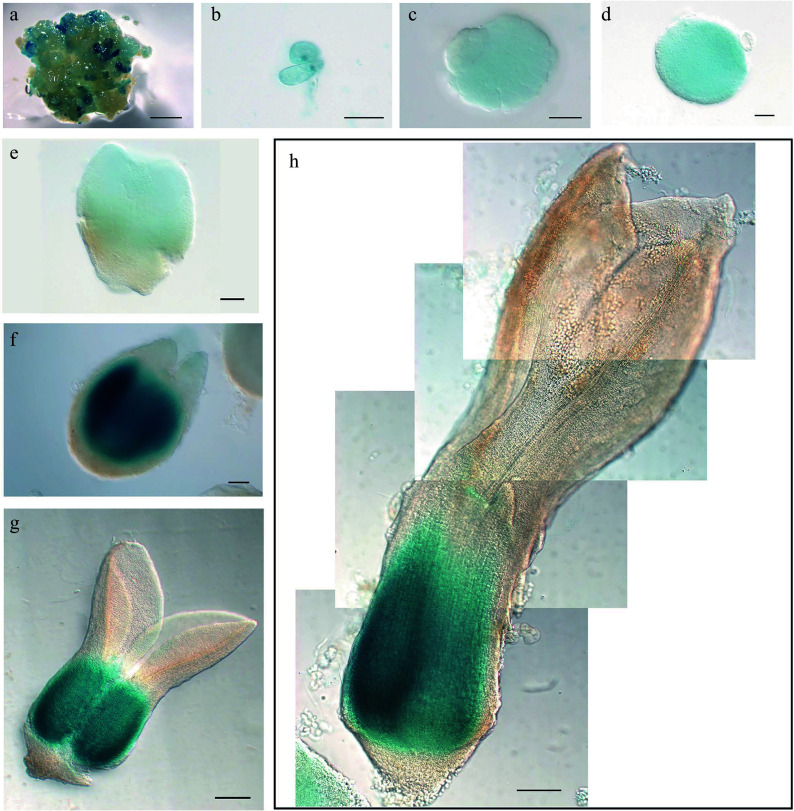
Expression pattern of *LcWOX9*_*pro*_: *GUS* during somatic embryogenesis in *Liriodendron*. (a) *LcWOX9*_*pro*_: *GUS* was very highly expressed in embryonic callus. (b) *LcWOX9*_*pro*_: *GUS* was expressed in single cells after 1 d on induction medium (IM). (c)–(e) *LcWOX9*_*pro*_: *GUS* was expressed in the (c) pre-globular embryo, the (d) globular embryo, and the (e) early heart-shaped embryo. (f) *LcWOX9*_*pro*_: *GUS* was expressed in a tissue-specific manner in the late heart-shaped embryo. (g) *LcWOX9*_*pro*_: *GUS* was highly expressed in the cotyledon embryo. (h) *LcWOX9*_*pro*_: *GUS* was expressed in a tissue-specific manner in the mature cotyledon embryo. Scale bars: (a) 2,000 μm; (b)–(e) 50 μm; (f) 100 μm; (g), (h) 200 μm.

The intermediate clade genes *WOX8* and *WOX9* are expressed very early in *Arabidopsis* embryo development ^[ 32] ^. We were only able to analyze expression of *LcWOX9* in *L. chinense*, as we were unable to identify a gene orthologous to *AtWOX8*. *LcWOX9* had the highest expression of any *WOX* gene in EC (Supplemental Fig. S2i), suggesting that it may be a critical regulator of embryonic properties. Despite a slight decrease in expression during the proembryo stage, *LcWOX9* showed consistent and robust expression throughout later SE developmental stages (Supplemental Fig. S2g). The *LcWOX9*_*pro*_: *GUS* reporter was highly expressed in EC ( Fig. 7a), and proembryo cells also had a robust *LcWOX9*_*pro*_: *GUS* signal ( Fig. 7b, c). At the late heart-shaped embryo and cotyledon embryo stages ( Fig. 7f, g), *LcWOX9*_*pro*_: *GUS* showed specific expression in the basal portion of the embryo, where it overlaped with expression of *LcWOX5*_*pro*_: *GUS* in the root tip ( Fig. 7h). This result suggests that *LcWOX9* may also participate in root meristem initiation and root development during later stages of SE. *LcWOX9* is a good candidate marker for SE in *Liriodendron* because of its extraordinarily high expression in EC.

### C. Expression patterns of the ancient clade genes *WOX13/14* during somatic embryogenesis

**Figure 8:**
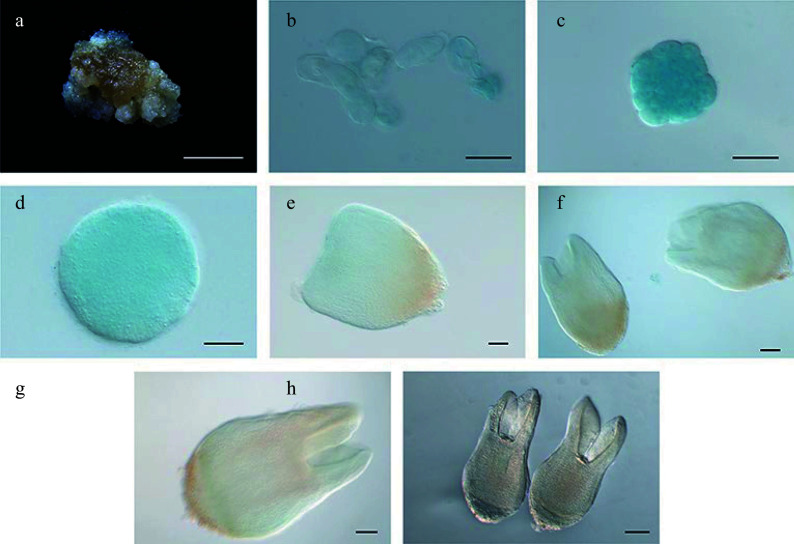
Expression pattern of *LcWOX14*_*pro*_: *GUS* during somatic embryogenesis in *Liriodendron*. (a) *LcWOX14*_*pro*_: *GUS* was not expressed in embryonic callus. (b), (c) *LcWOX14*_*pro*_: *GUS* was highly expressed in single cells after 1 d on (b) induction medium (IM) and in the (c) pre-globular embryo. (d)–(g) Expression of *LcWOX4*_*pro*_: *GUS* gradually declined in the (d) globular embryo, the (e) transition-stage embryo, the (f) late heart-shaped embryo, and (g) the torpedo embryo. (h) *LcWOX14*_*pro*_ *:GUS* was not expressed in the cotyledon embryo. Scale bars: (a) 2,000 μm; (b)–(e) 50 μm; (f), (g) 100 μm; (h) 200 μm.

*LcWOX13* and *LcWOX14* were the only ancient clade *WOX* genes identified in *L. chinense.* As mentioned above, *LcWOX13* expression was not detected at any stage of SE, whereas *LcWOX14* expression was consistent at several stages of SE (Supplemental Fig. S2h) and reached a maximum at the globular embryo stage. *LcWOX14*_*pro*_: *GUS*expression was detectable from the proembryo to the globular embryo stage ( Fig. 8a– d), but its signal was weak at the later torpedo stage ( Fig. 8e– h), suggesting that it may have a role in early SE stages. *Arabidopsis WOX14* and *WOX4* jointly regulate the development of vascular cells ^[ 33] ^, but *LcWOX14*_*pro*_: *GUS* and *LcWOX4*_*pro*_: *GUS* did not show similar expression patterns during SE of *Liriodendron* ( Fig. 7), and we did not observe specific expression of *LcWOX14*_*pro*_: *GUS* in the vasculature during SE ( Fig. 8). Therefore, it appears that *LcWOX14* may have distinct functions in the context of *Liriodendron* SE. Because *LcWOX13* failed to display clear expression during SE, an *LcWOX13* reporter line was not constructed.

## Discussion and conclusions

Growth and development of the model plant *Arabidopsis* are regulated by *WOX* genes, which exhibit distinct expression patterns throughout the plant life cycle. In this study, we used GUS reporters to investigate the expression patterns of *WOX* genes during SE in *L. chinense.*

In *Arabidopsis*, the transcription factor *AtWUS* maintains the stem cell niche by acting as an endogenous transcriptional activator and repressor. It is expressed in the OC of the shoot apical meristem ^[ 34] ^, where it plays a crucial role in stem cell maintenance. During early embryogenesis, *AtWUS* can be detected in the 16-cell embryo, and its expression remains restricted to a few cells throughout embryonic development ^[ 35] ^. Interestingly, *AtWOX5* shares structural and domain similarities with *AtWUS* ^[ 36] ^. *AtWOX5* is predominantly expressed in the QC, where it contributes to the regulation of stem cell populations in the root apical meristem (RAM) and columella to ensure cell number homeostasis ^[ 37] ^.

**Figure 9:**
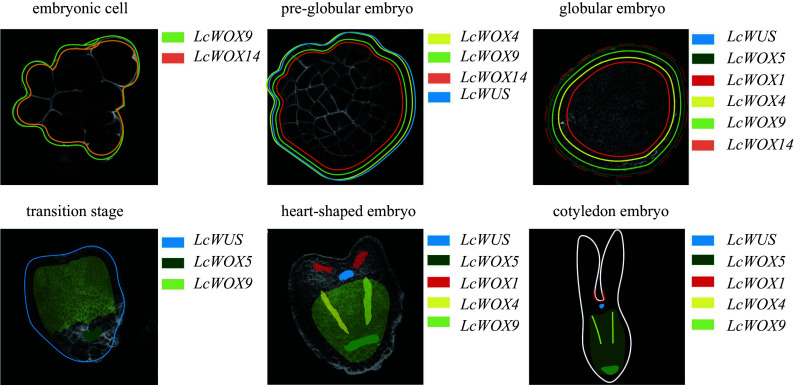
Diagrams illustrating the dynamic expression of *LcWOX* genes during somatic embryogenesis in *Liriodendron.*

*LcWOX5*_*pro*_: *GUS* and *LcWUS*_*pro*_: *GUS* signals were present during the globular embryo stage of *Liriodendron* SE but showed no spatial specificity ( Fig. 9). By contrast, *LcWUS*_*pro*_: *GUS* was specifically expressed in a small group of cells at the shoot apex at the torpedo embryo stage, much later than the heart-shaped embryo stage when *LcWOX5*_*pro*_: *GUS* was specifically expressed ( Fig. 9). The spatially localized expression of *LcWOX5*_*pro*_: *GUS* and *LcWUS*_*pro*_: *GUS* in our study occurred substantially later than that of their homologous genes during zygotic embryogenesis in *Arabidopsis* ^[ 14] ^, perhaps because the promoter sequenced we used lacked essential activation elements.

*WUS* plays a crucial role in embryogenesis by influencing cell fate and promoting embryo formation, and overexpression of WUS has been shown to induce SE ^[ 38] ^. This may explain, to some extent, why *LcWUS*_*pro*_: *GUS* was not highly expressed in embryogenic callus. The *LcWUS*_*pro*_: *GUS* expression pattern we observed indicated that *WUS* may maintain cell embryogenic capacity at a low but appropriate level during the early stages of SE. However, upregulation of *WUS* under specific treatments, such as hormone application, can promote SE.

In the present study, the expression pattern of *LcWOX5*_*pro*_: *GUS*in the root pole of *Liriodendron* appeared to be less specific than that of *Arabidopsis* *WOX5*. This difference may reflect the greater complexity of the shoot apical meristem (SAM), RAM, and other organ structures in *Liriodendron* compared with *Arabidopsis*. Thus, it will be necessary to confirm the structure of the *Liriodendron* RAM and the organization of different cell types in future work.

Nonetheless, promoter-driven reporter lines have limitations in accurately reflecting the spatial and temporal specificity of gene expression. For example, in *Arabidopsis* transgenic lines expressing GFP from an *AtWOX5*_*pro*_: *GFP* construct, GFP was observed alongside the QC and spread to a small number of adjacent cells in the RAM ^[ 29] ^. By contrast, expression of a *gWOX5*: *GFP* construct (containing an AtWOX5 genomic fragment) was restricted to the QC, indicating the presence of regulatory regions within the gene sequence, separate from the promoter region, that regulate spatiotemporal expression ^[ 39] ^.

The function of *LcWOX5* during the early stages of SE remains a subject of investigation. Previous studies have demonstrated that calli regenerated from leaves of *Arabidopsis* and rice express *WOX5*, suggesting that callus may possess root identity ^[ 9] ^. However, our qRT-PCR results revealed weak *LcWOX5* expression in EC, and the *LcWOX5*_*pro*_: *GUS* signal was barely detectable ( Fig. 3a & 4a). We therefore hypothesize that *LcWOX5* may promote the reprogramming of cell identity only during the transformation from callus to somatic embryo ^[ 40]^ but may have a limited effect on EC maintenance. Further investigations are required to fully characterize the precise functions and regulatory mechanisms of *LcWOX5* during SE.

*WOX4* is expressed mainly in vascular meristems, and WOX4-RNAi inhibits *WOX4* expression in *Arabidopsis*, resulting in much shorter transgenic plants, atrophy of phloem and xylem, and an increase in undifferentiated tissues ^[ 41] ^. Conversely, overexpression of *SlWOX4* in tomato leads to increased phloem and xylem formation. These findings imply that *WOX4* promotes proliferation and development of vascular stem cells ^[ 42] ^. In the present work, *LcWOX4* was expressed in the phloem throughout the later stages of SE development, suggesting that its role may be similar to those of its *WOX4* homologs ( Fig. 9), i.e., stimulating the proliferation and differentiation of vascular stem cells. Phloem and xylem formation are essential aspects of tree growth and forest improvement ^[ 43] ^, and *LcWOX4* will be a future focus for the improvement of key traits in forest trees.

Early embryo development is abolished in the *Arabidopsis* *wox8 wox9* double mutant, probably owing to abnormalities in cell division ^[ 44] ^, but no meristem defects were detected in the *wox9* single mutant at the seedling stage. Instead, *AtWOX9* plays a crucial role in the root, and lack of *AtWOX9* function may cause aberrant division of root meristem cells, resulting in the formation of short roots ^[ 45] ^. Likewise, *OsWOX9* controls the equilibrium of root apical meristem cells in rice ^[ 46] ^. Overexpression of *OsWOX9* prevents development of the root cap and leads to production of multi-stems and deformed leaves. *OsWOX9* is specifically expressed in the QC, where it is involved in maintaining the balance and specialization of apical stem cells ^[ 46] ^. Here, *LcWOX9*_*pro*_: *GUS* was precisely and highly expressed at the base of torpedo embryos, overlapping with the expression of *LcWOX5*_*pro*_: *GUS* ( Fig. 9). *LcWOX9* was also expressed in EC ( Fig. 9), suggesting that *LcWOX9* may have a role in initiation and maintenance of EC in *L. chinense*. Expression of *LcWOX9* was maintained at a high level throughout SE, and it may therefore be possible to develop *LcWOX9* as a marker for the early stage of SE in order to determine embryogenesis potential.

*LcWOX13* appeared not to be expressed during SE in *Liriodendron*, in contrast to the ubiquitous expression and involvement of *WOX13* in callus formation of *Arabidopsis*. However, the role of *WOX13* during somatic embryo regeneration remains unclear. In *Vitis* *vinifera,* three *VvWOX13* genes showed low expression in somatic embryos ^[ 47] ^, but in *Phoebe bournei*, two *PbWOX13* paralogs showed ubiquitous expression, with slightly increased expression of *PbWOX13a* during the later stages of somatic embryo development ^[ 48] ^. Here, *LcWOX14*_*pro*_: *GUS* was expressed from the somatic callus stage to the globular embryo stage with no significant change. In addition, we did not identify any *WOX11/12* orthologs in *Liriodendron*. *WOX11* has been reported to influence root system architecture and promote adventitious root formation during *de novo* root organogenesis from leaf explants ^[ 49] ^. These findings suggest that *LcWOX14* may play a role in the early stages of somatic embryo development, but whether it replaces the functions of both *LcWOX11/12* and *LcWOX13*, especially in root regeneration, remains to be determined.

*WOX2* and *WOX3* have been reported to participate in somatic embryo formation through direct activation by the totipotent transcription factor *LEC* ^[ 17]^ *.* However, we did not observe spatiotemporal specificity of *LcWOX2 _pro_*: *GUS* and *LcWOX3*_*pro*_: *GUS* expression, perhaps because of inadequate or defective activation.

In conclusion, *WOX* genes of different species show unique sub-functionalization and have acquired novel functions during the process of evolution, and it will be necessary to fully characterize the functions of individual *WOX* genes in *Liriodendron* in future research. It is also important to acknowledge that although SE can replicate certain developmental processes observed in zygotic embryogenesis, studies have revealed differences in the transcriptomes of zygotic and somatic embryos ^[ 21] ^. Therefore, it will be intriguing to explore the extent to which the molecular network that regulates zygotic embryogenesis can be extrapolated to the process of SE in different species ( Fig. 9).

## Materials and methods

### Plant materials, culture conditions, and plasmids

Hybridization between *L. chinense* and *L. tulipifera* was performed at the breeding orchard of Nanjing Forestry University, Xiashu (119.21E, 32.12N), Jiangsu Province, in late April 2015. Labeled immature aggregated samaras, which were generated by artificial pollination, were collected 8 weeks after pollination. Immature zygotic embryos with endosperm were immediately removed from samaras and transferred to callus induction medium (CIM). Embryogenic callus (genotype No. 154102) was initiated from immature embryos of hybrid *Liriodendron* and then maintained following sustained subculture. The procedure to induce hybrid *Liriodendron* somatic embryos has been described previously ^[ 19] ^. The pBI121( *35S*_*pro*_: *GUS*) vector was kindly provided by Professor Thomas Laux, Freiburg University, Germany. Promoter fragments of *LcWOX* genes were amplified from genomic DNA of *L. chinense* and cloned into the pBI121 binary vector to drive expression of the GUS gene. Details on promoter length are provided in Supplemental Table S1. The fragments were assembled into the *pBI121* vector by Gibson assembly (New England Biolabs, Beijing, NEB Cat. No. #E5510S) after the 35S promoter was removed by *Hind*III-HF (NEB Cat. No. #R3104S) and *Sma*I (NEB Cat. No. #R0141S) digestion. Promoter amplification primers and assembly primers are listed in Supplemental Table S1.

### *Agrobacterium*-mediated transformation

*A. tumefaciens* strain EHA105 harboring pBI121( *35S*_*pro*_: *GUS*) and modified pBI121 ( *LcWOX* promoter fragment driving GUS) binary vectors were used for transformation experiments. The strains were cultured on Luria–Bertani (LB) solid medium containing 25 mg·L ^−1^ rifampicin (Sigma, USA) and 50 mg·L ^−1^ kanamycin (Sigma, USA) and grown at 28 °C in the dark. The transformation protocol was identical to that reported previously ^[ 12] ^. For transgenic-positive selection, calli were recovered after *Agrobacterium* co-cultivation and incubated on callus selection medium (CSM) containing 90 mg·L ^−1^ G418 (Geneticin) to induce transgenic calli. Transgenic calli were sub-cultured once in a 25-d interval.

### Histological and histochemical analysis

For histological analysis of early-stage SE, samples were harvested after 1, 2–3, 4–5, 7, 8–9, 10, and 14 d on IM. Samples were incubated in an adequate amount of SRCI2200 ^[ 50]^ staining solution (0.1% SR2200 [v/v], 1% DMSO [v/v], 0.05% Trition-X100 [w/v], 5% glycerol [w/v], and 4% paraformaldehyde [w/v] in PBS buffer, pH 8.0) and vacuum treated for 15 min at room temperature. Afterwards, the samples were incubated in staining solution in the dark for one week. Before confocal microscopy imaging, the staining solution was carefully removed and replaced with an equal amount of PBS buffer to wash away excess dye. We detected SR2200 fluorescence following excitation with a 405-nm laser.

For histochemical analysis of GUS expression, the GUS signal was detected in transformed callus and embryos at different stages using a previously reported method with some modifications ^[ 51] ^. After fixing with pre-cooled 90% acetone for 30 min on ice, calli and embryos were stained with X-gluc staining solution (50 mM Na _2_PO _4_ buffer [pH 7.2], 5 mM K _3_Fe(CN) _6_, 5 mM K _4_Fe(CN) _6_, 0.5% Triton X-100 [v/v], 2 mM X-Glux) at 37 °C for 12 h in the dark. The staining process was terminated with 75% ethanol. After 2–3 rinses with Na _2_PO _4_ buffer, the samples were transferred to a clearing solution (chloral hydrate:glycerol:water, 8:1:2 [w/v/v]). The samples were destained and transparent after replenishment of the clearing solution (1–2 times).

### Phylogenetic analysis

WOX sequences from *Arabidopsis* were downloaded from The Arabidopsis Information Resource ( https://www.arabidopsis.org/), and WOX sequences from *S. lycopersicum*, *M. truncatula*, *P. trichocarpa*, *A. trichopoda*, *C. sativus* and *P. abies* were downloaded from http://planttfdb.cbi.pku.edu.cn ^[ 52] ^.

## SUPPLEMENTARY DATA

Supplementary data to this article can be found online.

## Supplementary Information

accompanies this paper at ( https://www.maxapress.com/article/doi/10.48130/FR-2023-0015)
