# Supplementary material for: Expression dynamics of WOX homeodomain transcription factors during somatic embryogenesis in Liriodendron hybrids
Source: For Res (Fayettev). 2023 Jun 13;3:15. doi: 10.48130/FR-2023-0015 (PMC11524298; doi:10.48130/FR-2023-0015)
Supplement: Supplementary file 1 — Supplementary data to this article can be found online. [file FR-2023-0015-S1.zip › 10.48130_FR-2023-0015-Suppl-FigureS2.pdf]

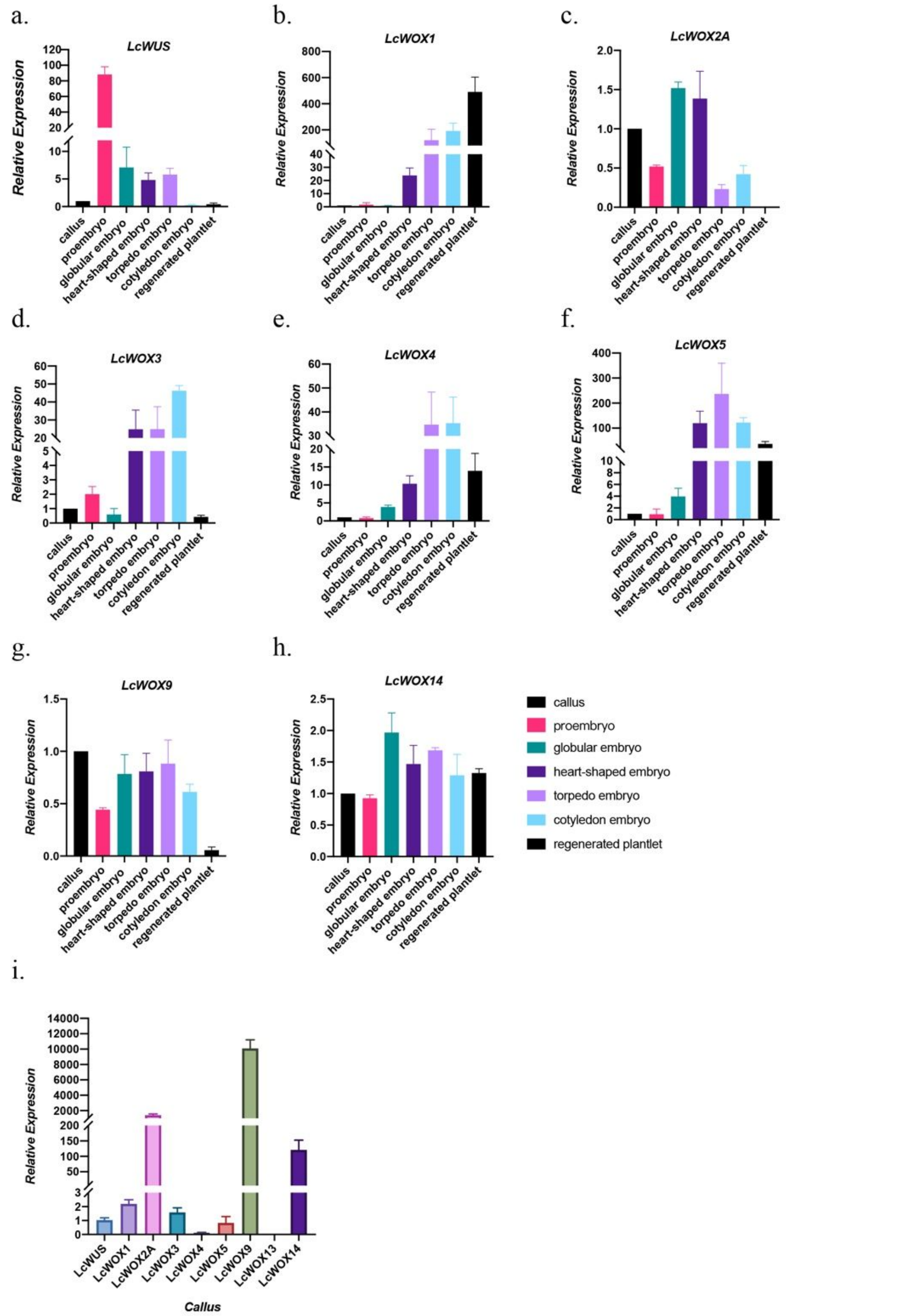

Figure S2. Relative expression of *LcWOX* genes during *Liriodendron* SE.

(a-h) Expression profiles of *LcWUS*, *LcWOX1*, *LcWOX2A*, *LcWOX3*, *LcWOX4*, *LcWOX5*, *LcWOX9*, and *LcWOX14* at different stages of somatic embryogenesis determined by qRT-PCR. (i) *LcWOX* genes show a variety of expression levels in embryonic callus. PCR signals were normalized to ubiqutin and 18S transcript levels. Data were acquired from three biological replicates.
