# Supplementary material for: Expression dynamics of WOX homeodomain transcription factors during somatic embryogenesis in Liriodendron hybrids
Source: For Res (Fayettev). 2023 Jun 13;3:15. doi: 10.48130/FR-2023-0015 (PMC11524298; doi:10.48130/FR-2023-0015)
Supplement: Supplementary file 1 — Supplementary data to this article can be found online. [file FR-2023-0015-S1.zip › 10.48130_FR-2023-0015-Suppl-FigureS3.pdf]

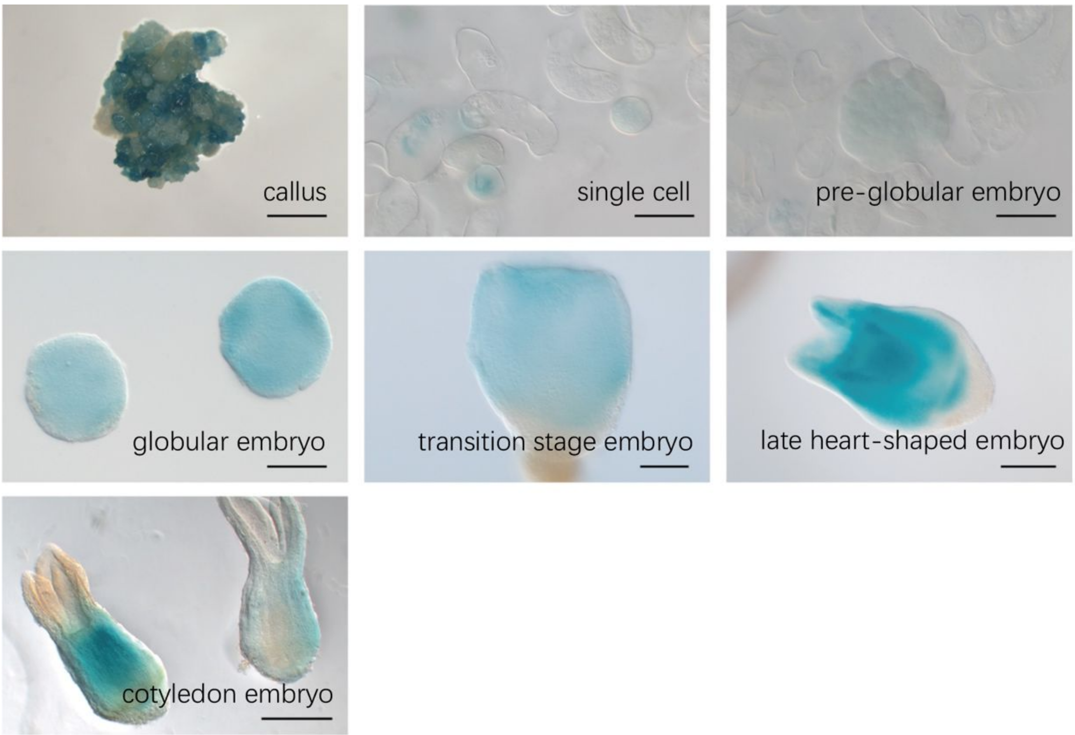

Figure S3. ***35S$_{pro}$*:GUS signals show no spatiotemporal specificity during SE of *Liriodendron.***

Scale bars: (a) 2 mm; (b–c) 50 µm; (d–e) 100 µm; (f) 2 mm; (g) 500 µm.
