# Supplementary material for: Expression dynamics of WOX homeodomain transcription factors during somatic embryogenesis in Liriodendron hybrids
Source: For Res (Fayettev). 2023 Jun 13;3:15. doi: 10.48130/FR-2023-0015 (PMC11524298; doi:10.48130/FR-2023-0015)
Supplement: Supplementary file 1 — Supplementary data to this article can be found online. [file FR-2023-0015-S1.zip › 10.48130_FR-2023-0015-Suppl-FigureS1.pdf]

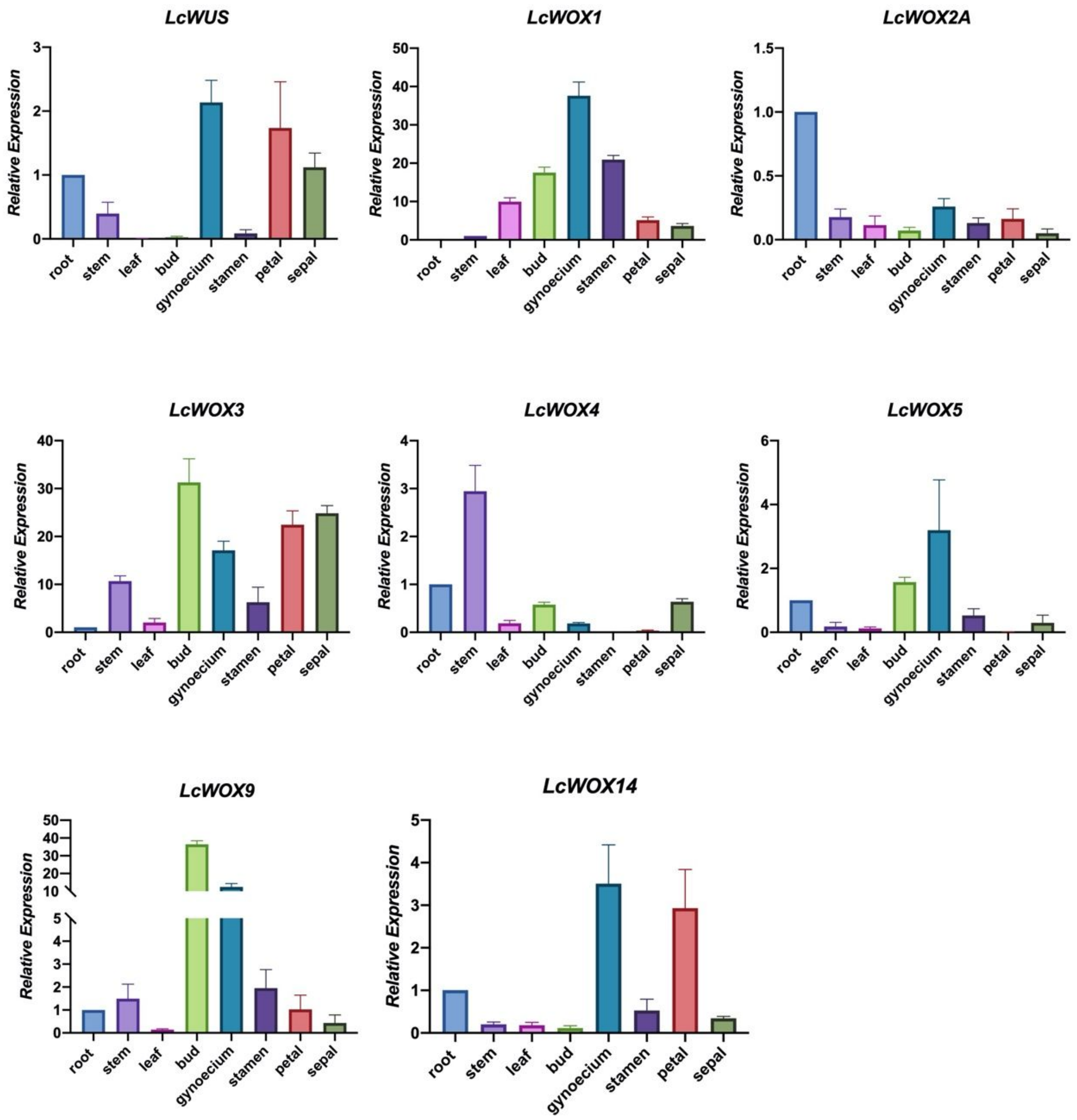
LcWUS
LcWOX1
LcWOX2A
LcWOX3
LcWOX4
LcWOX5
LcWOX9
LcWOX14
Relative Expression
root
stem
leaf
bud
gynoecium
stamen
petal
sepal

634

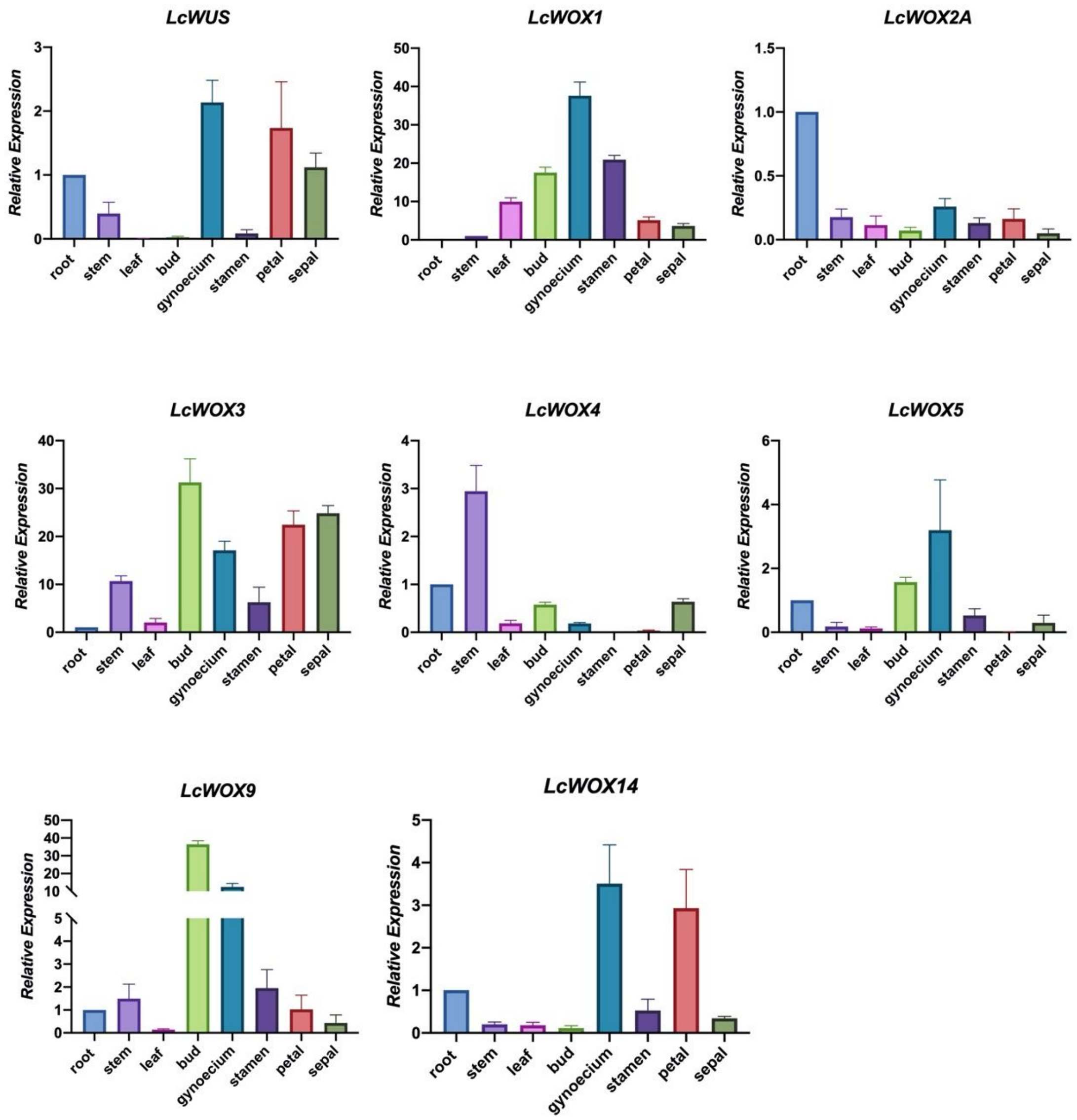

Figure S1. Expression profiles of *LcWUS*, *LcWOX1*, *LcWOX2A*, *LcWOX3*, *LcWOX4*, *LcWOX5*, *LcWOX9*, and *LcWOX14* in different tissues of *L. chinense* determined by qRT-PCR.

The PCR signals were normalized to ubiquitin and 18S transcript levels. Data were calculated using three replicates. Roots, stems, leaves, and leaf buds were harvested from 2-month-old regenerated seedlings. Gynoecium, stamen, petal, and sepal samples were collected from a mature tree (Lushan $1^{\#}$) growing in a breeding orchard in Xiashu.
